# A Novel Electrospinning Polyacrylonitrile Separator with Dip-Coating of Zeolite and Phenoxy Resin for Li-ion Batteries

**DOI:** 10.3390/membranes11040267

**Published:** 2021-04-08

**Authors:** Danxia Chen, Xiang Wang, Jianyu Liang, Ze Zhang, Weiping Chen

**Affiliations:** 1 School of Materials Science and Engineering, Wuhan University of Technology, Wuhan 430070, China; 234253@whut.edu.cn (D.C.); blzz@whut.edu.cn (Z.Z.); 290563@whut.edu.cn (W.C.); 2 Department of Mechanical Engineering, Worcester Polytechnic Institute, 100 Institute Road, Worcester, MA 01609, USA

**Keywords:** separator, electrospinning, polyacrylonitrile, zeolite, lithium-ion batteries

## Abstract

Commercial separators (polyolefin separators) for lithium-ion batteries still have defects such as low thermostability and inferior interface compatibility, which result in serious potential safety distress and poor electrochemical performance. Zeolite/Polyacrylonitrile (Z/PAN) composite separators have been fabricated by electrospinning a polyacrylonitrile (PAN) membrane and then dip-coating it with zeolite (ZSM-5). Different from commercial separators, the Z/PAN composite separators exhibit high electrolyte uptake, excellent ionic conductivity, and prominent thermal stability. Specifically, the Z/PAN-1.5 separator exhibits the best performance, with a high electrolyte uptake of 308.1% and an excellent ionic conductivity of 2.158 mS·cm^−1^. The Z/PAN-1.5 separator may mechanically shrink less than 10% when held at 180 °C for 30 min, proving good thermal stability. Compared with the pristine PAN separator, the Li/separator/LiFePO_4_ cells with the Z/PAN-1.5 composite separator have excellent high-rate discharge capacity (102.2 mAh·g^−1^ at 7 C) and favorable cycling performance (144.9 mAh·g^−1^ at 0.5 C after 100 cycles). Obviously, the Z/PAN-1.5 separator holds great promise in furthering the safety and performance of lithium-ion batteries.

## 1. Introduction

As a high-efficiency energy storage system, lithium-ion batteries have excellent performance such as greater energy density, high operational voltage, long cycle life, and low self-discharge rate. They have been widely used in mobile phones, intelligent speakers, notebook computers, electric scooters, and other portable electronic equipment [1,2,3,4,5,6]. With people’s increasing awareness of environmental protection and exhausted fossil oil, electric bicycles and electric vehicles have become the most promising industries. In recent years, incidents of spontaneous combustion and even explosion of mobile power supplies, electric bicycles, and electric vehicles have occurred one after another, which puts forward higher requirements for the safety of lithium-ion batteries [7,8,9,10]. Lithium-ion batteries usually are formed from cathodes, anodes, separators, and electrolytes. As a crucial constituent of the lithium-ion battery, the separator mainly plays a role in isolating cathodes and anodes and allows lithium ions to be transported freely in the lithium-ion battery [11,12]. The low melting point of polyolefin results in the bad thermal stability of polyolefin separators. At the same time, inferior interface compatibility between polyolefin separators and electrolytes leads to junior electrolyte wettability. Therefore, it is essential to develop a separator with prominent thermal stability and excellent wettability for lithium-ion battery applications [13,14].

In recent years, researchers have made extensive efforts to compensate for the shortcomings of polyolefin separators [15,16,17,18,19]. Electrospinning technology can be used to fabricate fibrous membranes with microporous and nanoporous structures. Electrospun fibrous membranes are widely used as separators due to their higher electrolyte absorption rate and excellent wettability with the electrolyte [20,21,22,23]. The commonly used polymers for preparing electrospinning membranes are polyvinylidene fluoride (PVDF) and its copolymers, polyimide (PI), polyacrylonitrile (PAN), etc. [24]. PVDF has a strong electron-withdrawing group (-C-F-), which is conducive to the ionization of lithium salt in the electrolyte, but its high degree of crystallinity will affect its ionic conductivity [20]. PI has good chemical stability and thermal stability due to its rigid aromatic ring and polar imide ring. It is a polymer with good comprehensive performance [25], but its high cost limits its application. The high melting point of PAN makes it have good thermal stability at high temperatures, and the nitrile group (-CN) in PAN is in favor of the dissociation of lithium ions in the electrolyte. Therefore, PAN is used to prepare separator skeletons by electrospinning due to its super thermal stability, good electrochemical properties, and reasonable price. The biggest problem in the application of fiber membranes in lithium-ion batteries is poor mechanical strength [26,27,28,29,30]. 

Phenoxy resin is a kind of thermoplastic resin, which does not need a curing agent in actual use. ZSM-5 is an aluminosilicate with uniform micropores and three-dimensional intersecting straight channels, with excellent thermal and chemical stability. Zeolites are mostly embedded by coating or blending [31]. Xiao et al. [32] prepared NaA zeolite-embedded polyethylene terephthalate (PET) nonwoven, and the thermal resistance and ionic conductivity were clearly improved due to the NaA zeolite-embedding. Li et al. [33] blended a polyimide (PI) polymer with a high melting point and zeolite (ZSM-5) to fabricate the zeolite/polyimide composite separator via a phase inversion process, with the purpose of enhancing the electrochemical properties and thermal stability of lithium-ion batteries. However, the addition of ZSM-5 significantly reduced the overall strength of the composite separator. It was difficult for ZSM-5 to disperse evenly in the casting liquid. Zhang et al. [34] prepared a zeolite/polyvinylidene fluoride (PVDF) composite separator by a phase inversion process. The result shows that the zeolite/PVDF composite separator exhibits excellent liquid electrolyte wettability, well-developed microstructure, and superior thermal resistance. The phase inversion process requires a large amount of solvent which is difficult to recycle. However, the above-mentioned composite separators made of non-woven fabrics, PI, or other polymers generally have insufficient mechanical strength, which will affect the safety of lithium-ion batteries under high-temperature conditions [35,36]. Phenoxy resin is used as an adhesive to improve the mechanical strength of composite separators because of its excellent mechanical properties and non-toxicity. In this study, a PAN fiber membrane was prepared with the electrospinning technique, phenoxy was introduced to improve the mechanical strength of PAN fiber membranes, and the addition of ZSM-5 was used to improve electrochemical performance. The PAN fiber membrane plays the role of backbone support to provide thermal stability for the separator, the phenolic resin acts as a binder to enhance the mechanical strength and ZSM-5 can improve electrolyte wettability of the composite separator. The results show that the composite separator has excellent mechanical strength and electrochemical properties. The influence of the concentration of ZSM-5 on the mechanical strength and ionic conductivity of the composite separator was researched and selected. Compared with the original PAN fiber membrane and PE separator, the Z/PAN-1.5 composite separator shows enhanced mechanical strength and electrochemical performance.

## 2. Materials and Methods

### 2.1. Materials

Polyacrylonitrile (PAN, purity ≥99.9%, M_w_ = 150,000) was provided by Hefei Sipin Technology Co., Ltd. (Hefei, China). N,N-Dimethylformamide (DMF) was bought from Shanghai Aladdin Biochemical Technology Co., Ltd. (Shanghai, China). Phenoxy resin was obtained from Guangzhou Jiabei Electronic Technology Co., Ltd. (Guangzhou, China). Acetone was purchased from Sinopharm Chemical Reagent Co., Ltd. (Beijing, China). Zeolite (ZSM-5) was supplied by Nankai University Chemical Reagent Factory. (Tianjin, China). Lithium iron phosphate (LiFePO_4_) was obtained from Taiwan Likai Power Technology Co., Ltd. (Taiwan, China). Acetylene black was purchased from Tianjin Yiborui Chemical Co., Ltd. (Tianjin, China). Polyvinylidene fluoride (PVDF) was bought from Arkema Fluor Chemical Co., Ltd. (Suzhou, China). The liquid electrolyte (1 mol·L^−1^ LiPF6, EC/EMC/DMC (1:1:1, volume ratio)) was supplied by Duoduo Chemical Technology Co., Ltd. (Suzhou, China). Commercial PE separator was purchased from SK Innovation for comparison (Changzhou, China).

### 2.2. Preparation of Electrospun PAN Separator

To prepare the solution for electrospun PAN separator preparation, PAN powder was dissolved in DMF under stirring at room temperature for 12 h to obtain a 10 wt% solution. The electrospinning equipment (ELITE) was provided by Beijing Yongkang Leye Technology Development Co., Ltd. (Beijing, China). Then, under a high voltage of 11 kV, enough solution was taken in the syringe and pushed out at a rate of 0.04 mm/min. During the spinning, the distance between the needle tip and collector was kept at 15 cm. After 11 h of spinning, the pure PAN separator was pressed at 120 °C for 30 min and dried at 80 °C for 12 h to extirpate the remaining solvent. The pure PAN separator is referred to as PAN in subsequent descriptions.

### 2.3. Preparation of Composite Separator

First, a certain amount of phenoxy resin was dissolved in the mixture of DMF/acetone (1:2, *v*/*v*) to obtain a 5 wt% phenoxy resin solution. To prepare the solution for dipping, varying amounts of ZSM-5 were dissolved in 5 wt% phenoxy resin solution under ultrasonic for 12 h. The concentrations of ZSM-5 (*w*/*v*) were 0%, 0.5%, 1%, 1.5%, and 2%, respectively. The previously prepared pure PAN separator was immersed in the ZSM-5 dispersion with different contents for 20 min and then taken out. The composite separators were first dried at room temperature until there was no obvious liquid on the surface and then dried in an oven at 60 °C for 12 h. For identification, composite partitions containing 0 wt%, 0.5 wt%, 1 wt%, 1.5 wt%, and 2 wt% ZSM-5 were defined as Z/PAN-0, Z/PAN-0.5, Z/PAN-1, and Z/PAN-1.5, Z/PAN-2, respectively.

### 2.4. Characterization of Composite Separator

In the range of 400–4000 cm^−1^, the Fourier transform infrared spectrum (FTIR, Nexus, Therno Nicolet, Waltham, MA, USA) was applied to demonstrate the presence of phenolic oxygen and ZSM-5 on the separator. The morphologies of composite separators were investigated by scanning electron microscopy (SEM, JSM-IT300, JEOL, Tokyo, Japan) after sputtering a thin layer of gold. The tensile strengths of the separator samples (50 mm × 10 mm) were tested by using a universal testing apparatus (Instron5967, Instron Pty Ltd., Norwood, MA, USA), at a speed of 5 mm·min^−1^. The contact angles of the separators and electrolytes were measured with a contact angle meter (OCA20, Dataphysics, Filderstadt, Germany). The electrolyte uptake of the separators was calculated by measuring the weight change before and after being soaked in the electrolyte for 2 h, and then calculated by the equation:

Electrolyte uptake (%) = (M − M_0_)/M_0_ × 100 (1)

where M_0_ is the weight of the separators without being soaked and M is the weight of the separators after being soaked in an electrolyte.

The thermal stability of the separators was analyzed by a thermogravimetric analyzer–differential scanning calorimeter (TGA–DSC, STA449F3, Netzsch, Selb, Germany) in the temperature range from 40 °C to 800 °C at a heating rate of 10 °C·min^−1^. The thermal shrinkage of separators was checked by placing membranes in a drying oven at different temperatures from 120 to 180 °C for 30 min. After being heated, the dimensional change was measured by the equation:

Shrinkage (%) = (A_0_ − A)/ A_0_ × 100 (2)

where A_0_ (cm^2^) and A (cm^2^) are the areas of the separator before and after being heated, respectively.

### 2.5. Electrochemical Performance of the Composite Separator

The composite separator and PE separator were stamped into discs with a diameter of 19 mm for use. The thickness of the PAN and composite separator used is 45 μm in the test. The electrochemical stability, ionic conductivity, and interfacial resistance (R_int_) were measured by using an electrochemical workstation (Ivium Stats, Ivium Technologies, Eindhoven, Netherlands). The thickness of the separator samples was measured with a thickness gauge (CH-1-S, Shanghai liuleng, China). The R_int_ between the separator sample and lithium metal electrode was carried out by electrochemical impedance spectroscopy (EIS). The ionic conductivity of the separators was also determined by electrochemical impedance spectroscopy (EIS), with two stainless steel electrodes. The tested frequency ranged from 0.1 Hz to 1 MHz with a signal amplitude of 5 mV. The ionic conductivity was calculated by the equation:

σ = d/(R × S)(3)

where d is the thickness of the separators sample, R is the bulk resistance and S is the area of the electrode. 

The electrochemical stability was measured in a cell of lithium metal/separator/ stainless steel by using liner sweep voltammetry from 2.5 V to 6.0 V at 5 mV·s^−1^. For rate performances and cyclability tests, a CR2032 coin-type cell was assembled by sandwiching the separator between a lithium anode and a LiFePO_4_ cathode and then adding a liquid electrolyte. The measurements were performed by battery-testing equipment (CT2001A, LAND Electronics, Wuhan, China). The cell was cycled at a fixed charge/discharge current density of 0.5 C for 100 cycles. Rate capability test was applied to the discharge of 0.2, 0.5, 1, 2, 3, 5, 7, and 0.2 C, under 2.5–4.2 V.

## 3. Results

**Figure 1:**
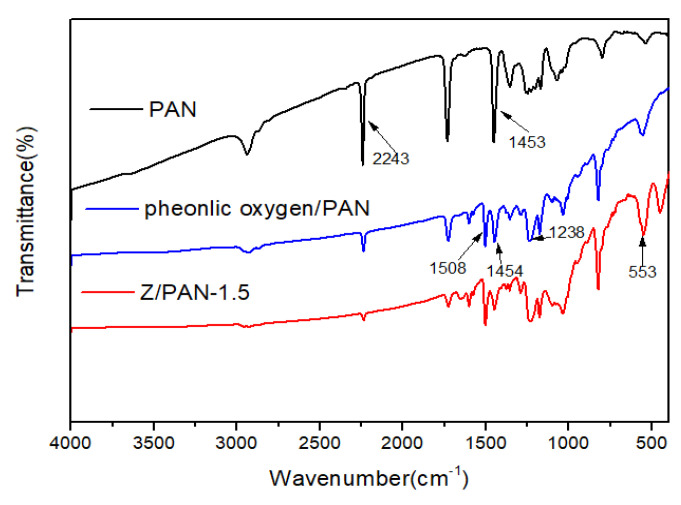
FT-IR spectra of electrospun PAN separator, phenoxy resin/PAN separator, and Z/PAN-1.5 separators.

FT-IR spectrums of the electrospun PAN separator, phenoxy resin/PAN separator, and Z/PAN-1.5 separators were recorded to confirm the smooth incorporation of phenoxy resin and ZSM-5 zeolite, as shown in Figure 1. In the infrared spectrum curve of PAN, the peak at 2243 cm^−1^ and 1453 cm^−1^ can be vested in the stretching vibration of the -CN bond and the bending vibration of -CH_2_- [29]. After immersing in 5 wt% phenoxy resin solution, a new peak appeared at 1238 cm^−1^, which is the characteristic absorption peak of the ether bond connected to the benzene ring, and the absorption peaks of the benzene skeleton at 1508 cm^−1^ and 1454 cm^−1^. Besides this, a T-O-T (T=Si or Al) bending vibration peak of ZSM-5 appeared at 553 cm^−1^, indicating that ZSM-5 is normally embedded into the Z/PAN-1.5 separators [33]. The above indicated that phenoxy resin and ZSM-5 were successfully introduced into the Z/PAN-1.5 separators.

**Figure 2:**
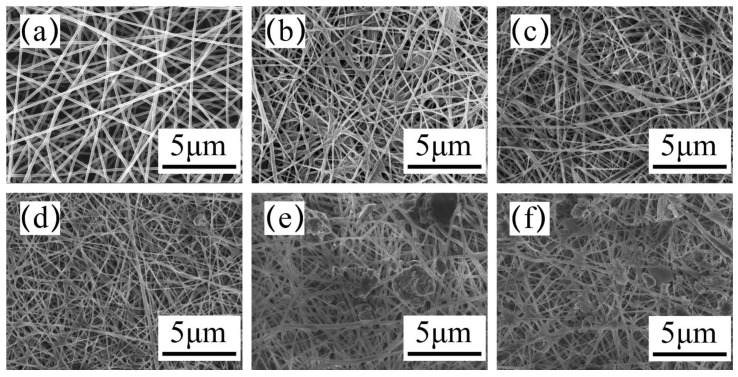
SEM micrographs for the surface of electrospun PAN separator and composite separator with different ZSM-5 concentrations: (**a**) PAN, (**b**) Z/PAN-0, (**c**) Z/PAN-0.5, (**d**) Z/PAN-1, (**e**) Z/PAN-1.5 and (**f**) Z/ PAN-2.

Figure 2 depicts the surface morphology of the electrospun PAN separator and different composite separators after immersing in 5 wt% phenoxy resin solution. It is observed in Figure 2a that the surface of PAN fiber is smooth and uniform without beads. According to Figure S1, it can be seen the average diameter of the fibers in the membrane is 180 nm. PAN fibers were simply lapped together to form a PAN separator after heat treatment. Although the large number of voids in the structure of PAN separators can better absorb the electrolyte, its poor mechanical properties cannot reach the minimum requirements for use. Figure 2b–f presents that phenoxy resin forms a certain bonding effect on the surface of PAN fibers, which helps to enhance the mechanical properties of PAN separators. As the concentration of ZSM-5 increases, the composite separator exhibits more and more particles. After embedding a few ZSM-5 particles into the composite separators, the separators’ absorption of the electrolyte is enhanced due to the multistage pore structure inside ZSM-5 nanoparticles [37].

**Figure 3:**
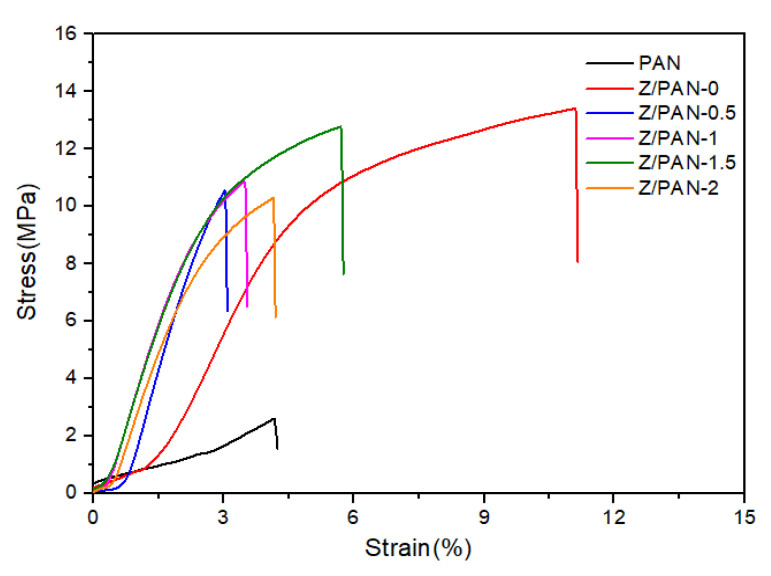
The stress–strain curves of different separators.

As depicted in Figure 3, the tensile strength of the composite separators that were immersed in the phenoxy resin solution was effectively improved. The tensile strength rose from 2.6 MPa for the PAN separator to 13.4 MPa for the Z/PAN-0 composite separator. The mechanical strength of the PAN separator is basically facilitated by the bonding of fibers to each other. Since the fibers were bonded by the addition of phenoxy resin at the laps together, slippage and breakage of the fibers were effectively prevented. At the same time, with the combination of ZSM-5, the tensile strength of the separator dropped a bit, but the tensile strength of the Z/PAN-1.5 separator was 13 Mpa, which qualified for practical battery applications.

**Figure 4:**
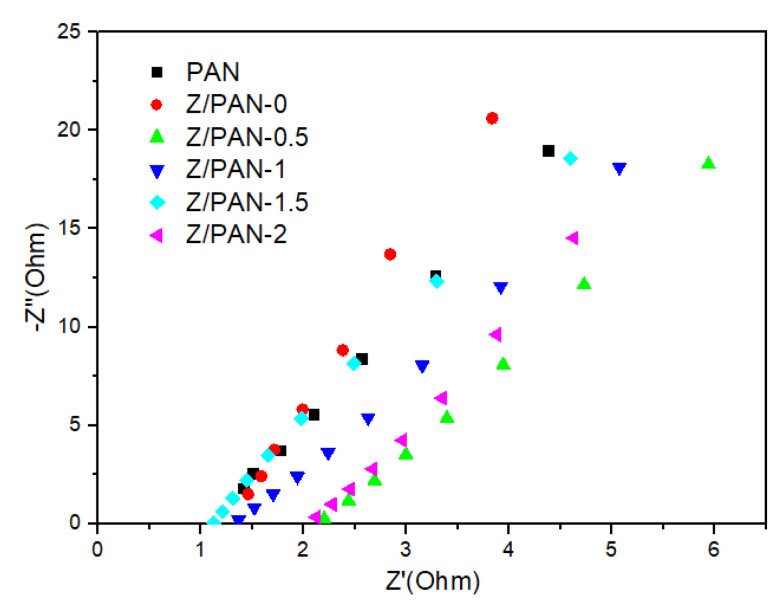
AC impedance spectra of the symmetric stainless-steel cells of different separators.

Figure 4 shows the Nyquist plot of the AC impedance of different liquid-electrolyte-soaked separators. In the high-frequency region, the intercept of the fitted curve on the real axis stands for the bulk resistance (R_b_). The R_b_ of PAN and Z/PANs (from 0 to 2) separators is 1.10, 1.27, 2.23, 1.42, 1.04, and 2.14 Ω, respectively. The ionic conductivity of PAN, Z/PAN-0 and Z/PAN-1.5 separators is 1.982 mS·cm^−1^, 1.712 mS·cm^−1^, 2.158 mS·cm^−1^ respectively. The ionic conductivity (Table 1) of the Z/PAN-1.5 separator is higher than that of the Z/PAN-0 separator, which is ascribed to the multistage pore structure inside ZSM-5 nanoparticles allowing more liquid electrolyte to be stored, as well as facilitating the migration of lithium-ion. Considering the satisfactory tensile strength and excellent ion conductivity, the Z/PAN-1.5 separator was chosen as a typical sample of the Z/PAN separators for succeeding studies.

**Figure 5:**
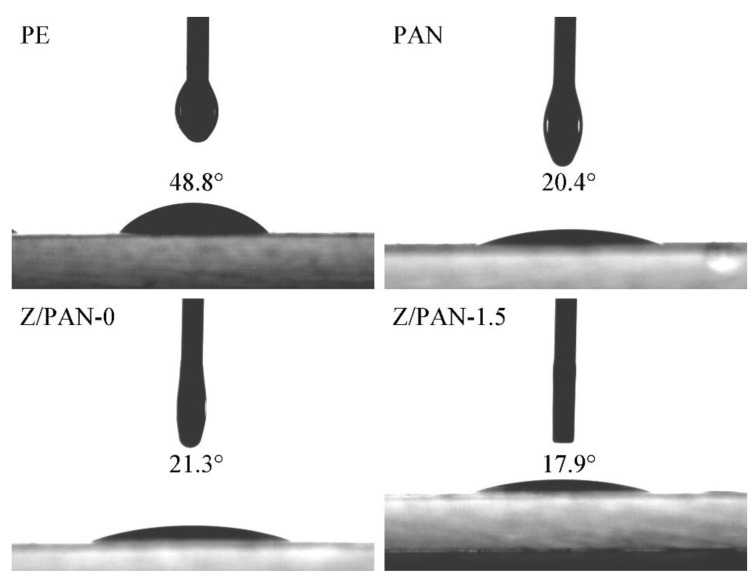
Digital photographs of contact angle of an electrolyte on PE separator, PAN separator, Z/PAN-0 separator, and Z/PAN-1.5 separator.

As listed in Table 1, the electrolyte uptake of the PAN separator is the highest at 385.7%, and those of the Z/PAN-0 separator and Z/PAN-1.5 separator are 195.2% and 308.1%, respectively. It can be seen that phenoxy resin has blocked part of the pores of the PAN separator, which reduces the porosity of the composite separator. However, the special micropore structure of ZSM-5 allows the composite separator to absorb more electrolyte, so the electrolyte uptake of the Z/PAN-1.5 separator has decreased a little. A separator with high wettability is essential for batteries, as it facilitates battery assembly in battery applications [38]. The contact angle of an electrolyte on different separators is exhibited in Figure 5. Generally, the smaller the contact angle the better the wettability of the separator. The PE separator shows a high electrolyte contact angle of 48.8°, while PAN and composite separators show improved electrolyte wettability. The contact angles of PAN, Z/PAN-0, and Z/PAN-1.5 separator are 20.4°, 21.3°, and 17.9°, respectively. The lower contact angles of the PAN separator and composite separators originate from their rough nanofiber surface.

**Figure 6:**
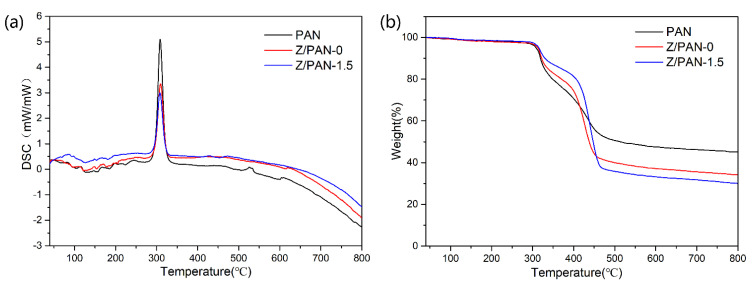
(**a**) DSC and (**b**) TG curves of the PAN separator, the Z/PAN-0 and Z/PAN-1.5 separator.

**Figure 7:**
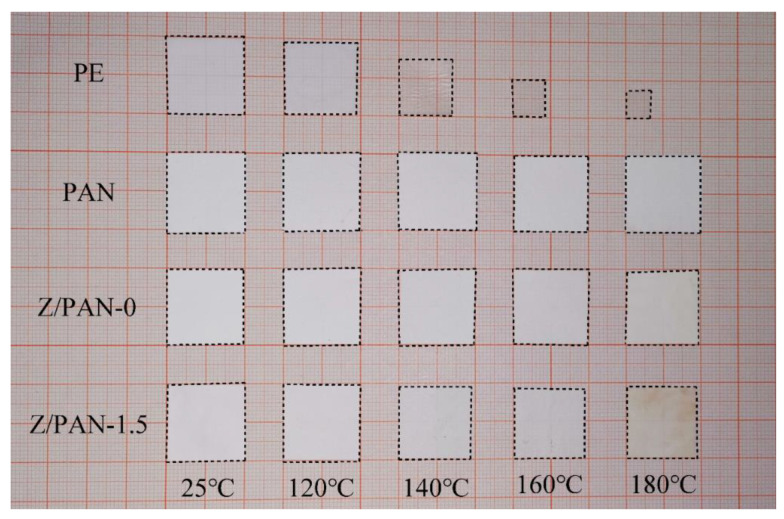
Thermal shrinkage of the separators observed at different temperatures.

The thermal stability of the separators is one of the important indicators of battery safety. A PE separator will shrink severely at high temperatures, causing a short circuit inside the battery and serious safety issues [39]. As presented in Figure 6a, the PAN separator, Z/PAN-0, and Z/PAN-1.5 separators show an endothermic peak at about 309 °C due to PAN melting. Among DSC curves, the PAN separator has a higher endothermic peak, because the molecular chain structure of PAN is more rigid than phenoxy resin, a PAN separator will absorb more heat per unit time when it reaches the decomposition temperature than the Z/PAN-0 separator. The melting temperature of PE separators is generally 130 °C which is much lower than that of PAN separators and composite separators. It can be seen from the results of the TG curves in Figure 6b that the quality of the three different separators begins to decrease significantly at around 300 °C. The quality of Z/PAN-0 and Z/PAN-1.5 separators decreases more slowly than that of the PAN separator during 300–400 °C. The temperature has not reached the decomposition temperature of the phenoxy resin structure, and the phenoxy resin is a certain obstacle in decomposition. With the temperature rising, the phenoxy resin gradually decomposes when reaching the decomposition temperature of the phenoxy resin, and the quality declines faster than before. To further investigate the thermal shrinkage of the composite separators, the separator samples are sandwiched by two glasses and then parked in an oven at a series temperature from 120 °C to 180 °C for 30 min. As displayed in Figure 7, The PE separator shrank acutely (88%) with its color varying from white to transparent, whereas the PAN separator basically did not change, and the composite separators varied little in size. The color of the Z/PAN-1.5 separator was only slightly yellow. Obviously, the excellent thermal stability of the composite separator is supposed to further the safety of the corresponding battery.

**Figure 8:**
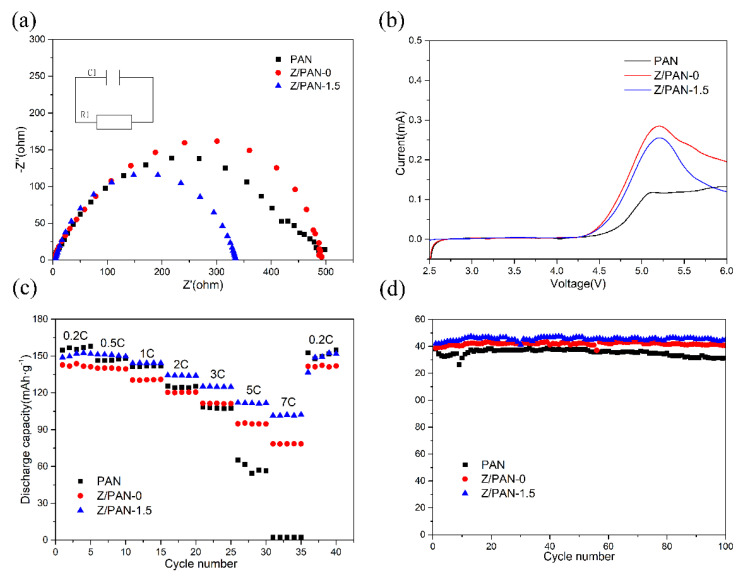
(**a**) The interfacial AC impedance spectra of cells assembled with different separators; (**b**) Linear scan voltammograms (LSV) curves of different separators; (**c**) Rate capability of the cells with different separators at the current densities range from 0.2 C to 7 C; (**d**) Cycle performance of the cells with different separators under 0.5 C.

Figure 8a depicts the equivalent circuit and the AC impedance spectra of different separators. It is clear that the Z/PAN-1.5 separators (about 331 Ω) show much smaller R_int_ than the PAN separator (about 439 Ω) and Z/PAN-0 separator (about 471 Ω). The smaller the R_int_, the better the interface compatibility. Obviously, the Z/PAN-1.5 separator has the best interface compatibility with electrodes. The reason should be attributed to the superior electrolyte wettability and preservation of the ZSM-5 embedded separator, which is conducive to favorable contact between the separator and electrodes. The electrochemical stability window of the separator is evaluated by linear sweep voltammetry as shown in Figure 8b. It is obvious that the electrochemical window of the Z/PAN-1.5 separator looks like that of the PAN separator, indicating that the Z/PAN-1.5 separator is stable enough to meet the battery applications. According to Figure 8c, the discharge capacities of the cells with different separators reduce gradually with the raising of discharge current density. The discharge capacities of cells using the PAN separator, Z/PAN-0 separator and Z/PAN-1.5 separator are 155.5 mAh·g^−1^, 141.2 mAh·g^−1^, and 152.5 mAh·g^−1^ at 0.2 C, respectively. It is evident that the Z/PAN-1.5 separator possesses much higher capacity retention than the PAN separator when the discharge current densities change from 0.2 C to 7 C. The specific discharge capacity of the Z/PAN-1.5 separator is 102.2 mAh·g^−1^, the Z/PAN-1.5 separator still maintains 67% of the discharge capacity at 0.2 C when the discharge current densities cycle at 7 C. At the same time, the Z/PAN-1.5 separator almost possesses little capacity. Figure 8d proves the cycling performance of batteries combined with different separators at 0.5 C. It can be seen that the Z/PAN-1.5 separator exhibits better cycle stability up to 100 cycles with little degradation when cycled at a permanent current density of 0.5 C. The superior rate performance and cycling performance of the Z/PAN-1.5 separator demonstrate its high feasibility for actual battery applications.

## 4. Conclusions

In this paper, electrospinning technology is used to prepare a PAN separator. After phenoxy resin and ZSM-5 treatment, a Z/PAN-1.5 separator with a tensile strength of 13 MPa is prepared. Due to the excellent thermal stability of the PAN matrix and ZSM-5, the Z/PAN-1.5 separator exhibits satisfactory thermal durability, which is good for the safety of batteries. Simultaneously, it also has superb electrolyte wettability and is low interface resistance. More importantly, the battery assembled with the Z/PAN-1.5 separator shows stable cycle performance and excellent rate performance. In general, the novel composite separator can function as an excellent performance separator, a good substitute for actual Li-ion batteries.

## Figures and Tables

**Table 1:** The ionic conductivity, electrolyte uptake, and porosity of different separators soaked with electrolytes at room temperature.

	Ionic Conductivity, mS·cm^−1^	Electrolyte Uptake, %	Porosity, %
PE	0.592	45.2	41.4
PAN	1.982	385.7	73.2
Z/PAN-0	1.712	195.2	55.5
Z/PAN-1.5	2.158	308.1	68.3

## Data Availability

The data presented in this study are available on request from the corresponding author.

## Supplementary Materials

The following are available online at https://www.mdpi.com/article/10.3390/membranes11040267/s1, Figure S1: The size distribution of membrane fiber.
