# Supplementary material for: A Novel Electrospinning Polyacrylonitrile Separator with Dip-Coating of Zeolite and Phenoxy Resin for Li-ion Batteries
Source: Membranes (Basel). 2021 Apr 8;11(4):267. doi: 10.3390/membranes11040267 (PMC8068060; doi:10.3390/membranes11040267)
Supplement: Supplementary file 1 [file membranes-11-00267-s001.pdf]

**Supplementary Materials:**

The temperature is maintained at 30 °C and the humidity is between 40% and 50% during electrospinning. The thickness of the PAN and composite separator used is 45μm in the test. The average diameter of the fibers in the membrane is 180 nm.

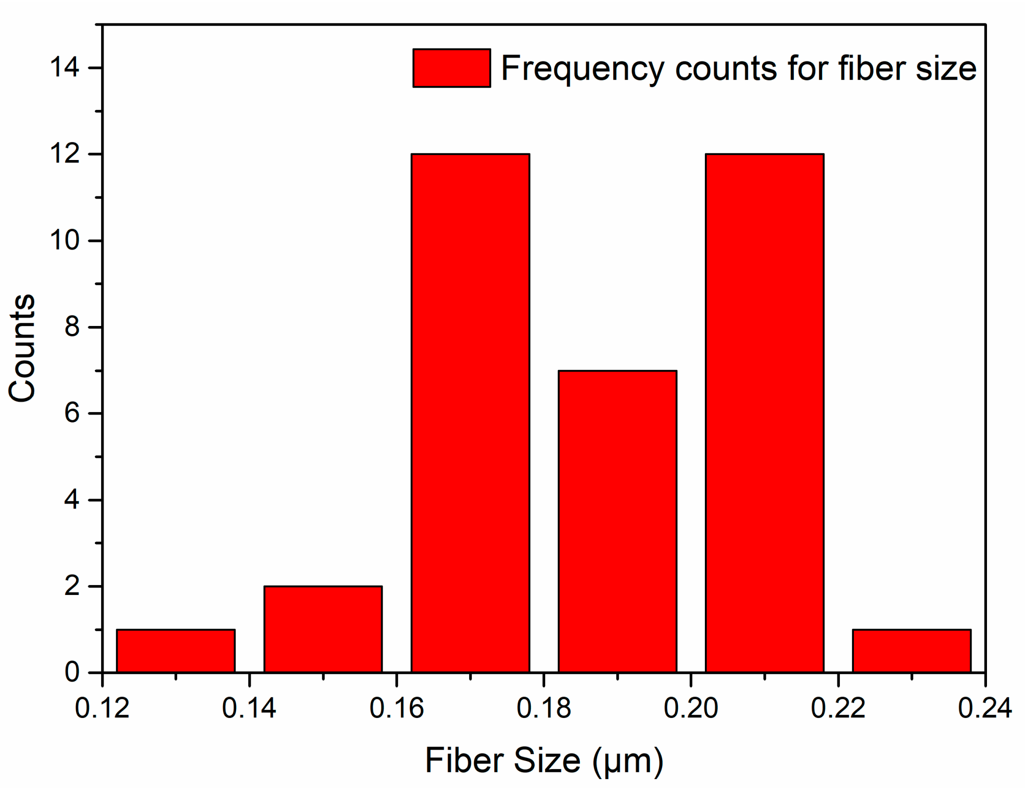


**Figure S1.** The size distribution of membrane fiber.
